# Supplementary material for: DNAJB1-PRKACA in HEK293T cells induces LINC00473 overexpression that depends on PKA signaling
Source: PLoS One. 2022 Feb 15;17(2):e0263829. doi: 10.1371/journal.pone.0263829 (PMC8846505; doi:10.1371/journal.pone.0263829)
Supplement: S1 Fig — (a) DNA gels used to determine the successful CRISPR deletion. Expected PCR product size for A and B clones was 551bp and for G clones was 615bp. (b) Blots demonstrate the expression of PKA-Cα and DP fusion protein in engineered HEK-DP clones as presented in Fig 1. (c) CREB immunoblot in A9, A11, and HEK-WT performed in triplicate. (d) Phosphorylated CREB in A9, A11, and HEK-WT. (e) α-PKA immunoblot of A9 cells following siRNA treatment (right panel) and corresponding GAPDH immunoblot (left panel). (PDF) [file pone.0263829.s001.pdf]

Supplementary Figure S1.

(a)

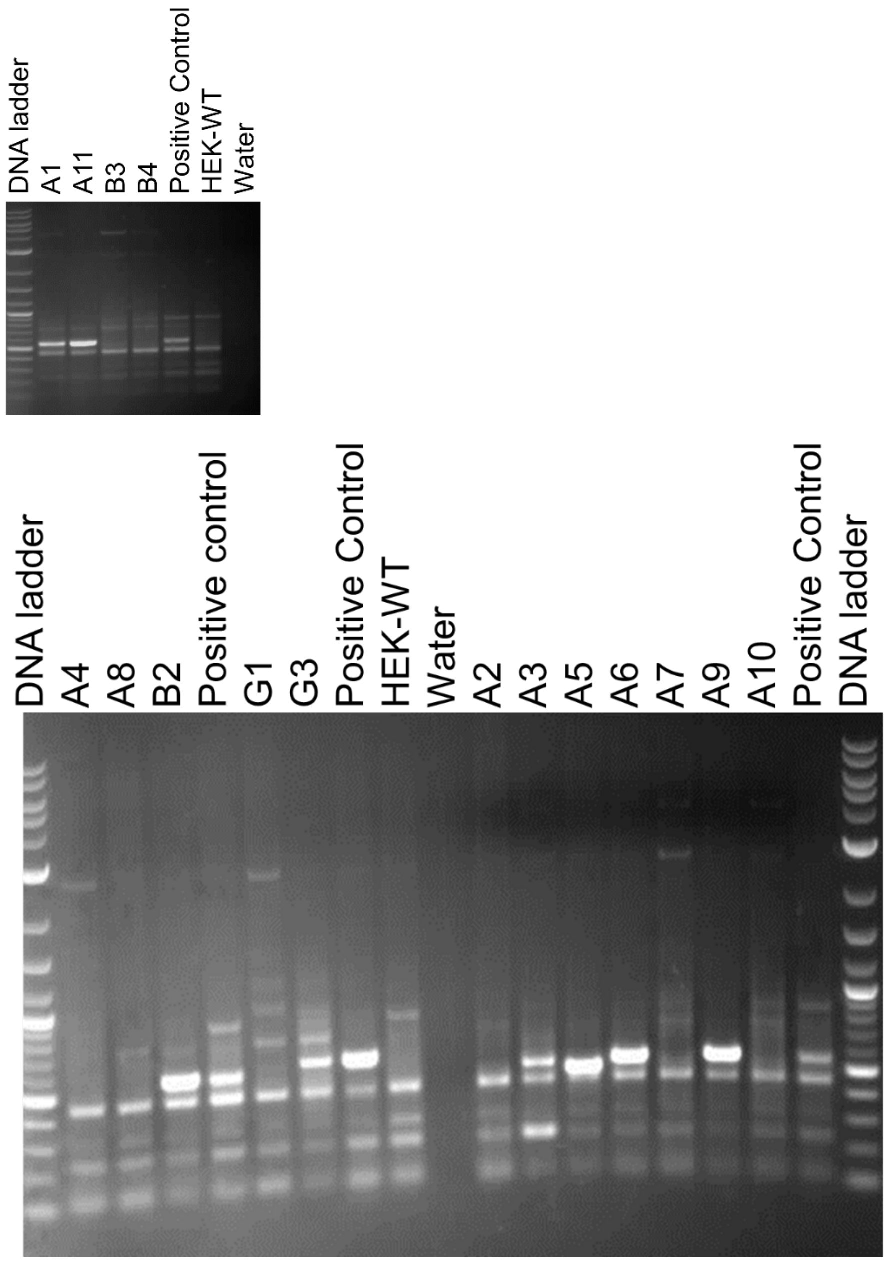

(b)

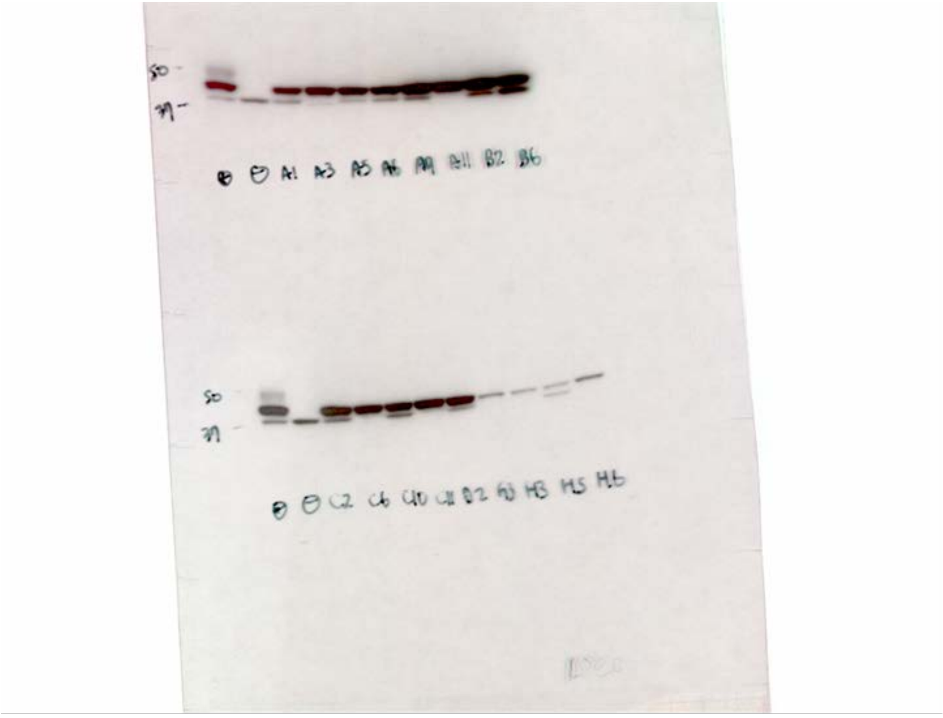

(c)

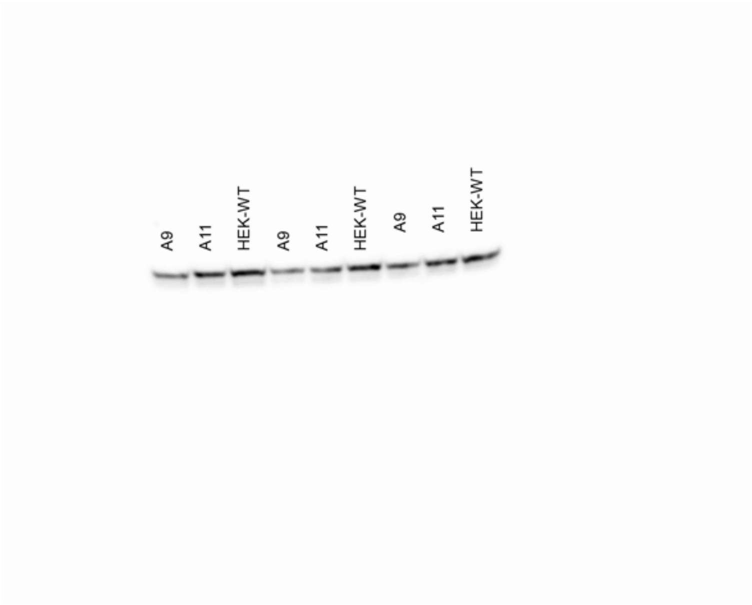

(d)

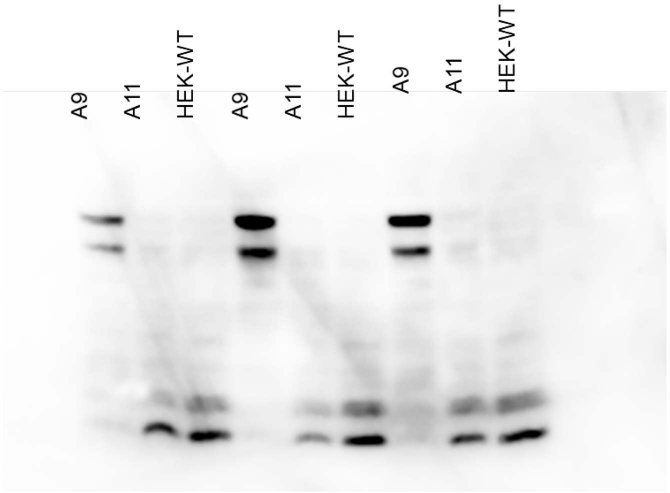

(e)

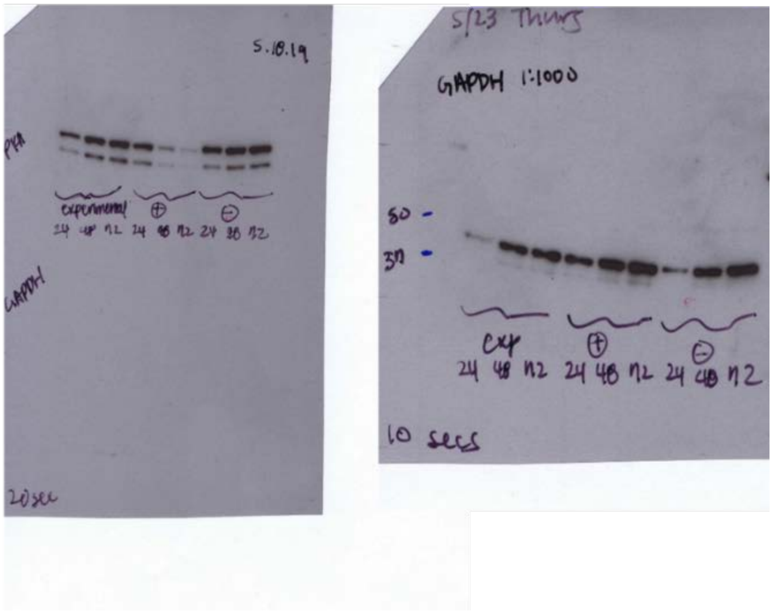

Supplementary Figure S1. Full-length blots. (a) DNA gels used to determine the successful CRISPR deletion. Expected PCR product size for A and B clones was 551bp and for G clones was 615bp. (b) Blots demonstrate the expression of PKA-Cα and DP fusion protein in engineered HEK-DP clones as presented in Figure 1. (c) CREB immunoblot in A9, A11, and HEK-WT performed in triplicate. (d) Phosphorylated CREB in A9, A11, and HEK-WT. (e) α–PKA immunoblot of A9 cells following siRNA treatment (right panel) and corresponding GAPDH immunoblot (left panel).
