# Supplementary material for: DNAJB1-PRKACA in HEK293T cells induces LINC00473 overexpression that depends on PKA signaling
Source: PLoS One. 2022 Feb 15;17(2):e0263829. doi: 10.1371/journal.pone.0263829 (PMC8846505; doi:10.1371/journal.pone.0263829)
Supplement: S2 Fig — Five HEK-CT clones which were established after transfection with vectors lacking guide RNA sequences have the same level of LINC00473 expression compared to HEK-WT cells. (PDF) [file pone.0263829.s002.pdf]

Supplementary Figure S2.

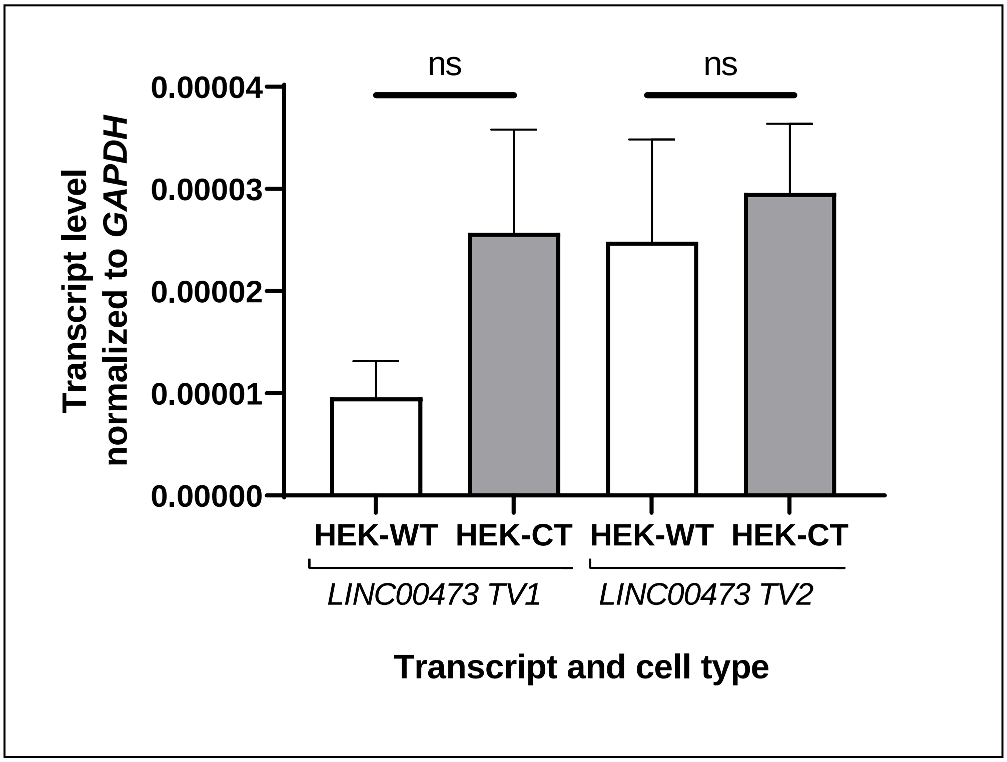

Supplementary Figure S2. *LINC00473* expression is unchanged in HEK-CT cells compared to HEK-WT cells. Five HEK-CT clones which were established after transfection with vectors lacking guide RNA sequences have the same level of *LINC00473* expression compared to HEK-WT cells.
