# Supplementary material for: DNAJB1-PRKACA in HEK293T cells induces LINC00473 overexpression that depends on PKA signaling
Source: PLoS One. 2022 Feb 15;17(2):e0263829. doi: 10.1371/journal.pone.0263829 (PMC8846505; doi:10.1371/journal.pone.0263829)
Supplement: S3 Fig — (a) Hsp70 expression is demonstrated in all cell lines. Following co-immunoprecipitation with PKA-Cα antibody, Hsp70 is identified in the protein complexes from A9 and A11. (b) Full-length blot represented in panel A. (PDF) [file pone.0263829.s003.pdf]

Supplementary Figure S3.

(a)

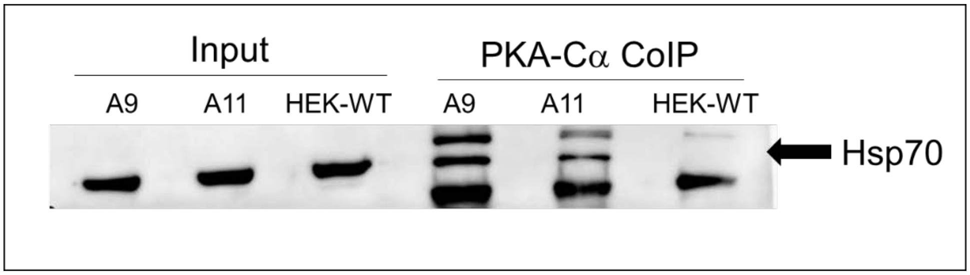

(b)

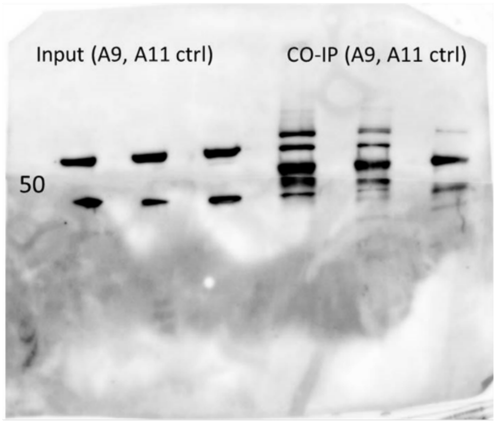

Supplementary Figure S3. Hsp70 interacts with PKA-Cα in A9 and A11 cells. (a) Hsp70 expression is demonstrated in all cell lines. Following co-immunoprecipitation with PKA-Cα antibody, Hsp70 is identified in the protein complexes from A9 and A11. (b) Full-length blot represented in panel A.
