# Supplementary material for: DNAJB1-PRKACA in HEK293T cells induces LINC00473 overexpression that depends on PKA signaling
Source: PLoS One. 2022 Feb 15;17(2):e0263829. doi: 10.1371/journal.pone.0263829 (PMC8846505; doi:10.1371/journal.pone.0263829)
Supplement: S4 Fig — (a) Bag2 protein co-immunoprecipitates with DP and PKA-Cα using PKA antibody. (b) DP and PKA immunoprecipitate using BAG2 antibody. (c) Full-length blots represented in a and b panels. GAPDH immunoreactivity is present in both input blots but not in the immunoprecipitated samples. (PDF) [file pone.0263829.s004.pdf]

Supplementary Figure S4.

(a)

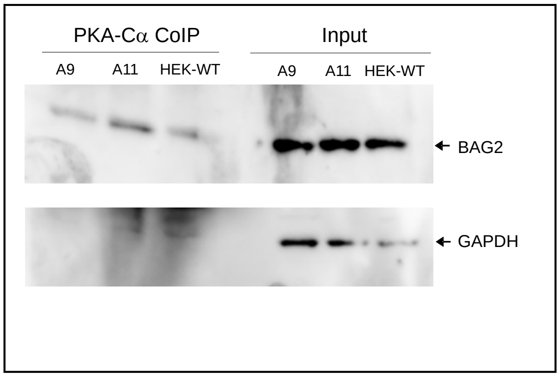

(b)

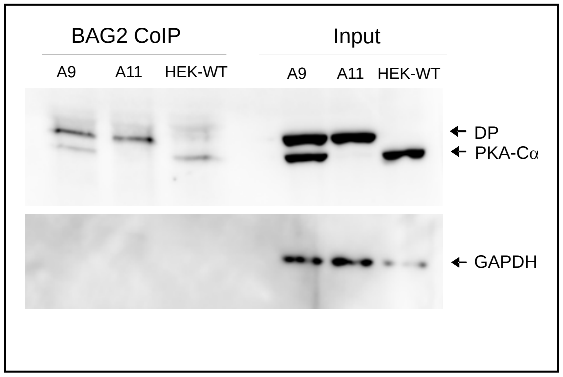

(c)

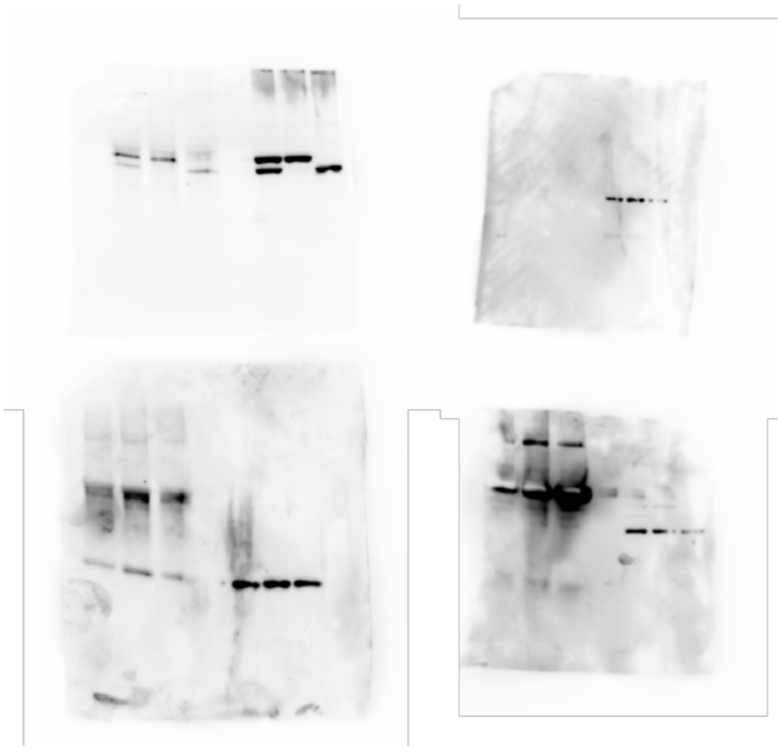

Supplementary Figure S4. BAG2 interacts with DP and PKA-Cα. (a) Bag2 protein co-immunoprecipitates with DP and PKA-Cα using PKA antibody. (b) DP and PKA immunoprecipitate using BAG2 antibody. (c) Full-length blots represented in a and b panels. GAPDH immunoreactivity is present in both input blots but not in the immunoprecipitated samples.
