# Supplementary material for: DNAJB1-PRKACA in HEK293T cells induces LINC00473 overexpression that depends on PKA signaling
Source: PLoS One. 2022 Feb 15;17(2):e0263829. doi: 10.1371/journal.pone.0263829 (PMC8846505; doi:10.1371/journal.pone.0263829)
Supplement: S1 Table — (PDF) [file pone.0263829.s005.pdf]

Supplementary Table S1.

Gene	Protein	A9 vs. HEK-WT<br>Adj. p-value	A11 vs. HEK-WT<br>Adj. p-value
DNAJB1	DnaJ homolog subfamily B member 1	3.09E-06	8.73E-07
CTSH	Pro-cathepsin H	3.09E-06	8.73E-07
PRKACA	cAMP-dependent protein kinase catalytic subunit alpha	3.09E-06	8.73E-07
RSRC2	Arginine/serine-rich coiled-coil protein 2	3.09E-06	8.73E-07
XRN2	5-3 exoribonuclease 2	3.09E-06	8.73E-07
RPL9	60S ribosomal protein L9	3.09E-06	8.73E-07
GANAB	Neutral alpha-glucosidase AB	3.09E-06	8.87E-07
ACSL3	Long-chain-fatty-acid--CoA ligase 3	3.26E-06	8.73E-07
EMC7	ER membrane protein complex subunit 7	3.32E-06	8.73E-07
MYO1C	Unconventional myosin-Ic	3.32E-06	8.73E-07
CFL1	Cofilin-1;Cofilin-2	3.32E-06	8.73E-07
LIMA1	LIM domain and actin-binding protein 1	3.32E-06	8.73E-07
PFDN2	Prefoldin subunit 2	3.32E-06	8.73E-07
AAAS	Aladin	3.32E-06	9.47E-07
BAG2	BAG family molecular chaperone regulator 2	3.4E-06	1.12E-06
LUC7L3	Luc7-like protein 3	3.5E-06	1.04E-06

LRPPRC	Leucine-rich PPR motif-containing protein, mitochondrial	3.65E-06	1.45E-06
COPA	Coatomer subunit alpha;Xenin;Proxenin	3.65E-06	1.13E-06
DSG2	Desmoglein-2	3.65E-06	8.73E-07
DBN1	Drebrin	3.65E-06	1.03E-06
RPL12	60S ribosomal protein L12	3.97E-06	1.94E-06
H3F3B	Histone H3;Histone H3.2;Histone H3.1t;Histone H3.3;Histone H3.1;Histone H3.3C	3.97E-06	1.45E-06
LETM1	LETM1 and EF-hand domain-containing protein 1, mitochondrial	4.02E-06	1.51E-06
MYH10	Myosin-10	4.86E-06	1.34E-06
GNB2	Guanine nucleotide-binding protein G(I)/G(S)/G(T) subunit beta-2;Guanine nucleotide-binding protein subunit beta-4	5.1E-06	2.12E-06
DNAJB11	DnaJ homolog subfamily B member 11	5.41E-06	1.94E-06
ATP5O	ATP synthase subunit O, mitochondrial	7.39E-06	2.64E-06
PHGDH	D-3-phosphoglycerate dehydrogenase	8.22E-06	2.72E-06
TBL2	Transducin beta-like protein 2	2.35E-05	8.02E-06
WDR6	WD repeat-containing protein 6	2.89E-05	1.08E-05

Supplementary Table S1. List of potential proteins which interact with DP fusion protein based on proteomic analysis.
