# Supplementary material for: DNAJB1-PRKACA in HEK293T cells induces LINC00473 overexpression that depends on PKA signaling
Source: PLoS One. 2022 Feb 15;17(2):e0263829. doi: 10.1371/journal.pone.0263829 (PMC8846505; doi:10.1371/journal.pone.0263829)
Supplement: S2 Table — Ranked pathways of significance based on differential protein precipitation in response to PKA-Cα immunoprecipitation in A9 and A11 compared to HEK-WT. (PDF) [file pone.0263829.s006.pdf]

Supplementary Table S2.

Pathway Term	Description	p-value	FDR
Metabolism of proteins Homo sapiens R-HSA-392499	Metabolism of proteins Homo sapiens R-HSA-392499	3.012E-06	0.005
double-stranded RNA binding (GO:0003725)	double-stranded RNA binding (GO:0003725)	6.8601E-05	0.007
tRNA binding (GO:0000049)	tRNA binding (GO:0000049)	6.6877E-05	0.007
mRNA binding (GO:0003729)	mRNA binding (GO:0003729)	7.1708E-05	0.007
translation factor activity, RNA binding (GO:0008135)	translation factor activity, RNA binding (GO:0008135)	6.3133E-05	0.007
single-stranded RNA binding (GO:0003727)	single-stranded RNA binding (GO:0003727)	6.5609E-05	0.007
snoRNA binding (GO:0030515)	snoRNA binding (GO:0030515)	6.1527E-05	0.008
rRNA binding (GO:0019843)	rRNA binding (GO:0019843)	6.1131E-05	0.008
telomerase RNA binding (GO:0070034)	telomerase RNA binding (GO:0070034)	6.1131E-05	0.008
primary miRNA binding (GO:0070878)	primary miRNA binding (GO:0070878)	6.0737E-05	0.008
siRNA binding (GO:0035197)	siRNA binding (GO:0035197)	6.0345E-05	0.009
AU-rich element binding (GO:0017091)	AU-rich element binding (GO:0017091)	6.0345E-05	0.009
miRNA binding (GO:0035198)	miRNA binding (GO:0035198)	6.0345E-05	0.009
piRNA binding (GO:0034584)	piRNA binding (GO:0034584)	5.9956E-05	0.01
snRNA binding (GO:0017069)	snRNA binding (GO:0017069)	5.9956E-05	0.01
G-quadruplex RNA binding (GO:0002151)	G-quadruplex RNA binding (GO:0002151)	5.9568E-05	0.01
pre-miRNA binding (GO:0070883)	pre-miRNA binding (GO:0070883)	5.9568E-05	0.01
RNA cap binding (GO:0000339)	RNA cap binding (GO:0000339)	5.9568E-05	0.015
RNA stem-loop binding (GO:0035613)	RNA stem-loop binding (GO:0035613)	5.9183E-05	0.01
pre-mRNA binding (GO:0036002)	pre-mRNA binding (GO:0036002)	5.9183E-05	0.01
histone pre-mRNA DCP binding (GO:0071208)	histone pre-mRNA DCP binding (GO:0071208)	5.9183E-05	0.01
BRE binding (GO:0042835)	BRE binding (GO:0042835)	5.9183E-05	0.02
telomeric repeat-containing RNA binding (GO:0061752)	telomeric repeat-containing RNA binding (GO:0061752)	5.9183E-05	0.02

7S RNA binding (GO:0008312)	7S RNA binding (GO:0008312)	5.9183E-05	0.02
21U-RNA binding (GO:0034583)	21U-RNA binding (GO:0034583)	5.88E-05	0.02
misfolded RNA binding (GO:0034336)	misfolded RNA binding (GO:0034336)	5.88E-05	0.02
base pairing with RNA (GO:0000498)	base pairing with RNA (GO:0000498)	5.88E-05	0.03
actin filament binding (GO:0051015)	actin filament binding (GO:0051015)	0.00028692	0.03
ribonuclease P RNA binding (GO:0033204)	ribonuclease P RNA binding (GO:0033204)	5.88E-05	0.03
N6-methyladenosine-containing RNA binding (GO:1990247)	N6-methyladenosine-containing RNA binding (GO:1990247)	5.88E-05	0.04
alpha-aminoacyl-tRNA binding (GO:1904678)	alpha-aminoacyl-tRNA binding (GO:1904678)	5.88E-05	0.05
actin lateral binding (GO:0003786)	actin lateral binding (GO:0003786)	0.00063334	0.06
RNA binding (GO:0003723)	RNA binding (GO:0003723)	5.88E-05	0.06

Supplementary Table S2. Gene enrichment pathway analysis based on proteomic data. Ranked pathways of significance based on differential protein precipitation in response to PKA-Cα immunoprecipitation in A9 and A11 compared to HEK-WT.
